# Supplementary material for: Identifying the distinct roles of dual dopants in stabilizing the platinum-nickel nanowire catalyst for durable fuel cell
Source: Nat Commun. 2024 Jan 13;15:508. doi: 10.1038/s41467-024-44788-0 (PMC10787824; doi:10.1038/s41467-024-44788-0)
Supplement: Supplementary file 1 — Supplementary Information [file 41467_2024_44788_MOESM1_ESM.pdf]

Supporting Information for

Identifying the distinct roles of dual dopants in stabilizing the platinum-nickel nanowire catalyst for durable fuel cell

Lei Gao[1,§], Tulai Sun[2,§], Xuli Chen[1,§], Zhilong Yang[1], Mengfan Li[1], Wenchuan Lai[1], Wenhua Zhang[3], Quan Yuan[1] and Hongwen Huang[1,4]✉

[1]College of Materials Science and Engineering, State Key Laboratory of Chemo/Biosensing and Chemometrics, Hunan University, Changsha, Hunan 410082, P. R. China.

[2]Center for Electron Microscopy, State Key Laboratory Breeding Base of Green Chemistry Synthesis Technology and College of Chemical Engineering, Zhejiang University of Technology, Hangzhou, Zhejiang 310014, P. R. China.

[3]Department of Chemical Physics, University of Science and Technology of China, Hefei, Anhui 230026, P. R. China.

[4]Shenzhen Research Institute of Hunan University, Shenzhen, Guangdong 518055, P. R. China.

[§]These authors contributed equally: Lei Gao, Tulai Sun, Xuli Chen.

✉Correspondence and requests for materials should be addressed to H. H. (huanghw@hnu.edu.cn).

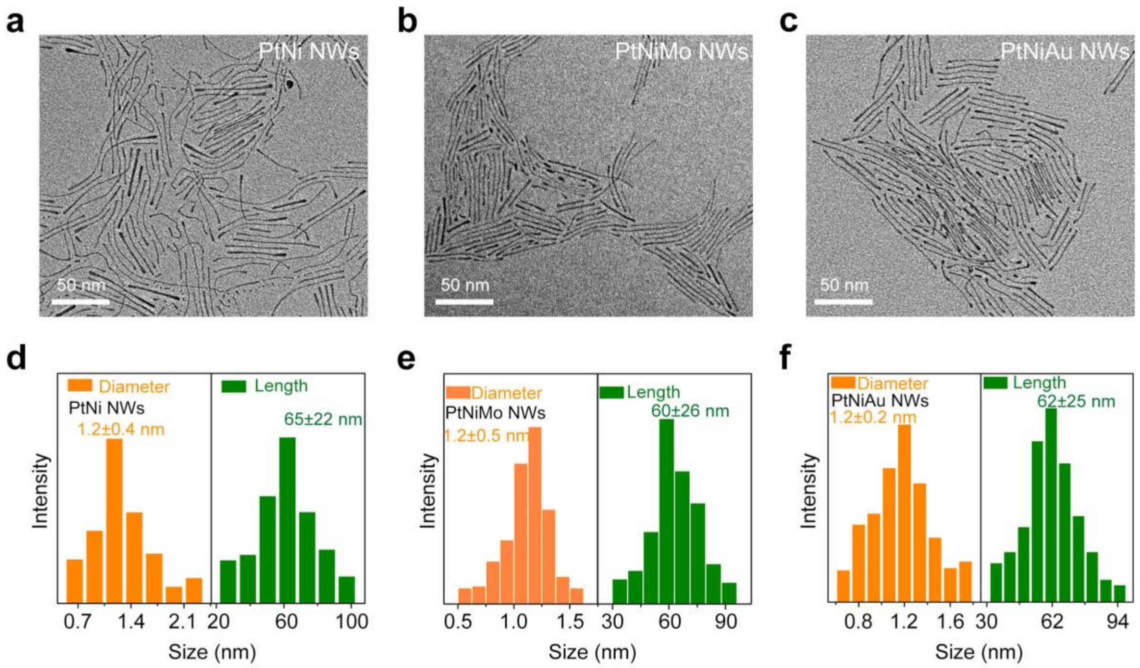

Supplementary Figure 1. TEM image of (a) PtNi NWs, (b) PtNiMo NWs and (c) PtNiAu NWs. Size distribution for (d) PtNi NWs, (e) PtNiMo NWs and (f) PtNiAu NWs.

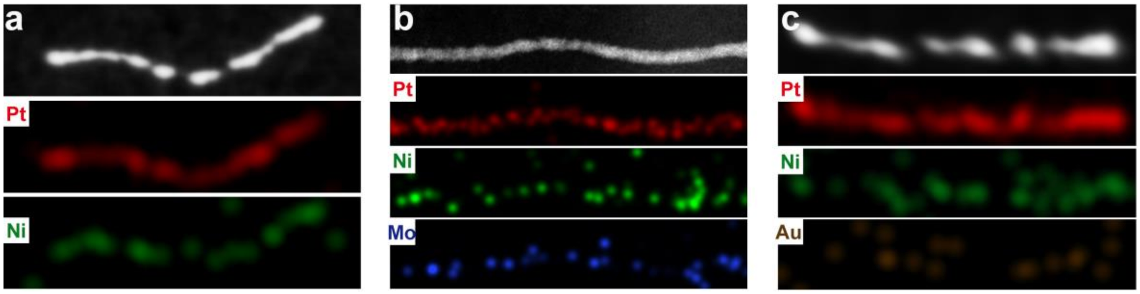

Supplementary Figure 2. EDS element mapping of (a) PtNi NWs, (b) PtNiMo NWs and (c) PtNiAu NWs.

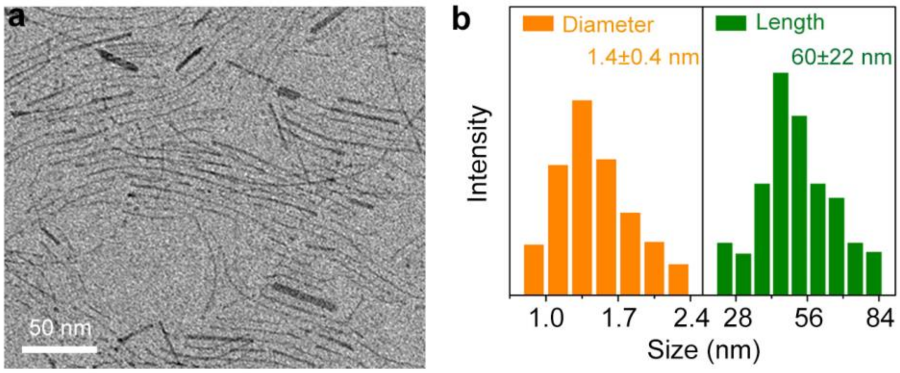

Supplementary Figure 3. (a) TEM image and (b) size distribution for Pt NWs.

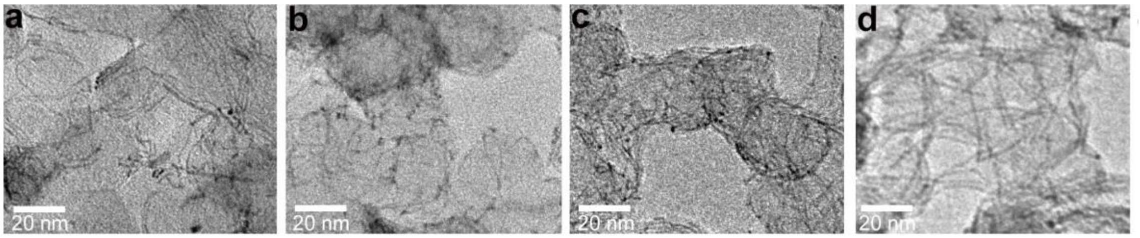

Supplementary Figure 4. TEM images of (a) Pt NWs/C, (b) PtNi NWs/C, (c) PtNiMo NWs/C, and (d) PtNiAu NWs/C catalysts before ADTs.

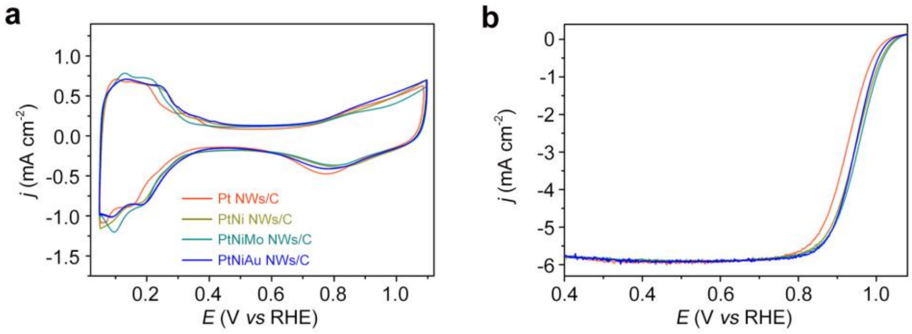

Supplementary Figure 5. a) CVs recorded at room temperature in N_2-saturated 0.1 M $HClO_4$ solutions at a sweep rate of 50 mV s^{-1}. b) ORR polarization curves recorded at room temperature in O_2-saturated 0.1 M $HClO_4$ solutions at a sweep rate of 10 mV s^{-1} and a rotation rate of 1600 rpm.

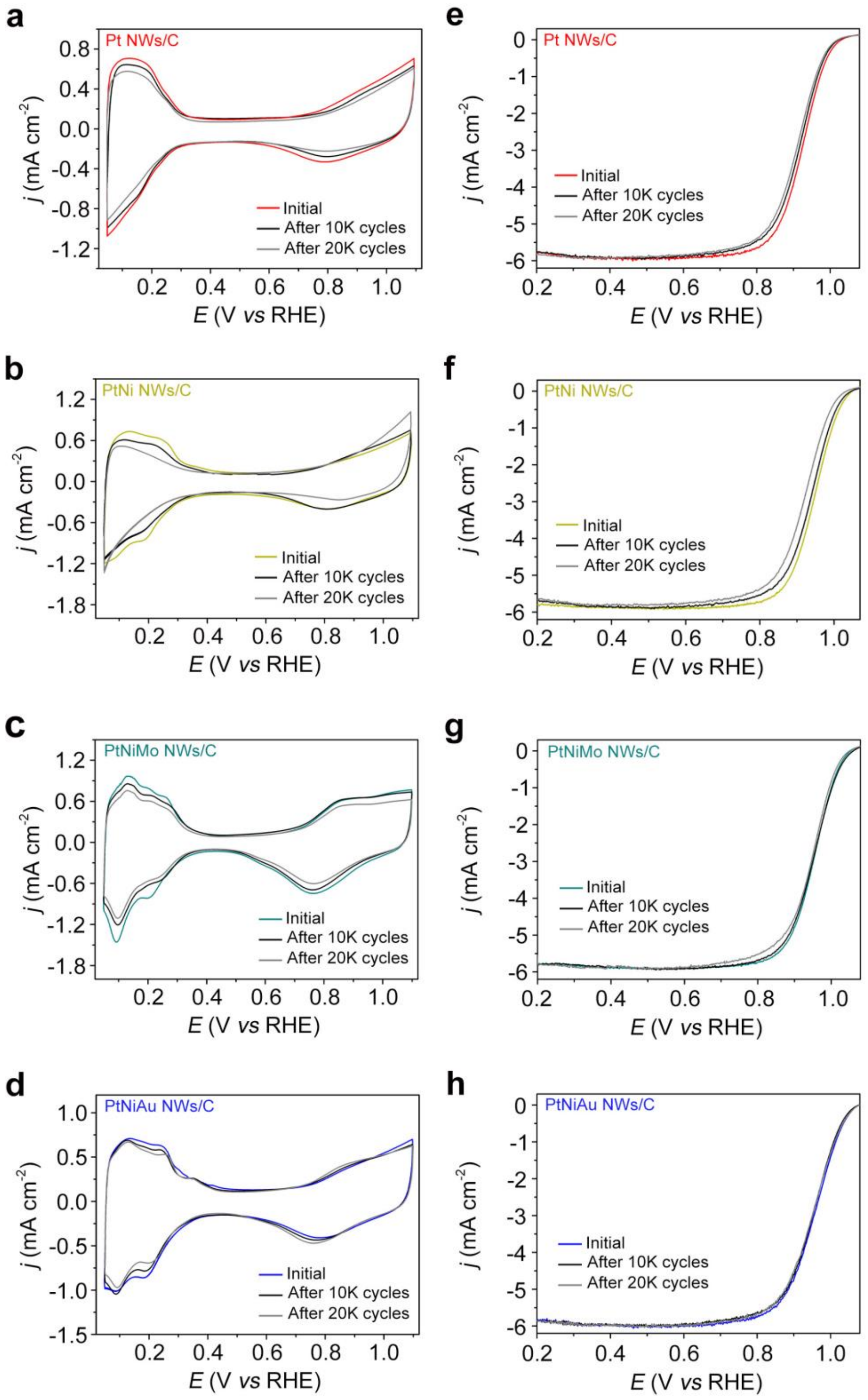

Supplementary Figure 6. a-d) CV curves and e-h) ORR polarization curves of Pt NWs/C, PtNi NWs/C, PtNiMo NWs/C, and PtNiAu NWs/C catalysts before and after ADT of different cycles.

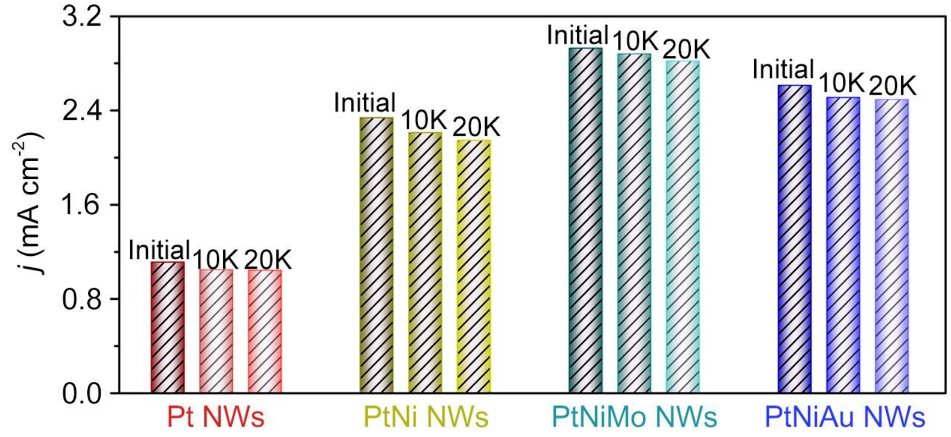

Supplementary Figure 7. The specific activity of Pt NWs/C, PtNi NWs/C, PtNiMo NWs/C, and PtNiAu NWs/C catalysts before and after ADT of different cycles.

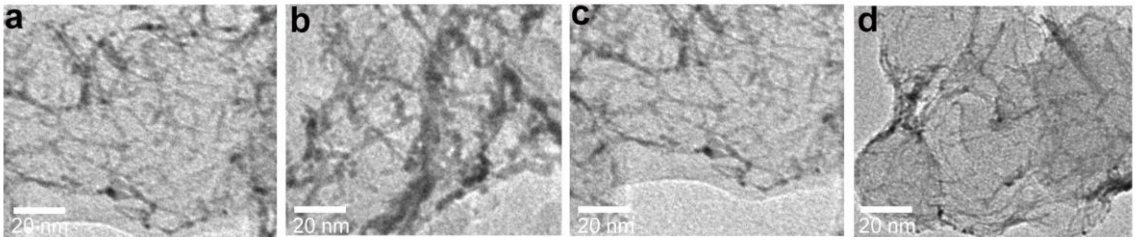

Supplementary Figure 8. TEM images of (a) Pt NWs/C, (b) PtNi NWs/C, (c) PtNiMo NWs/C, and (d) PtNiAu NWs/C catalysts after 20K cycles of ADT.

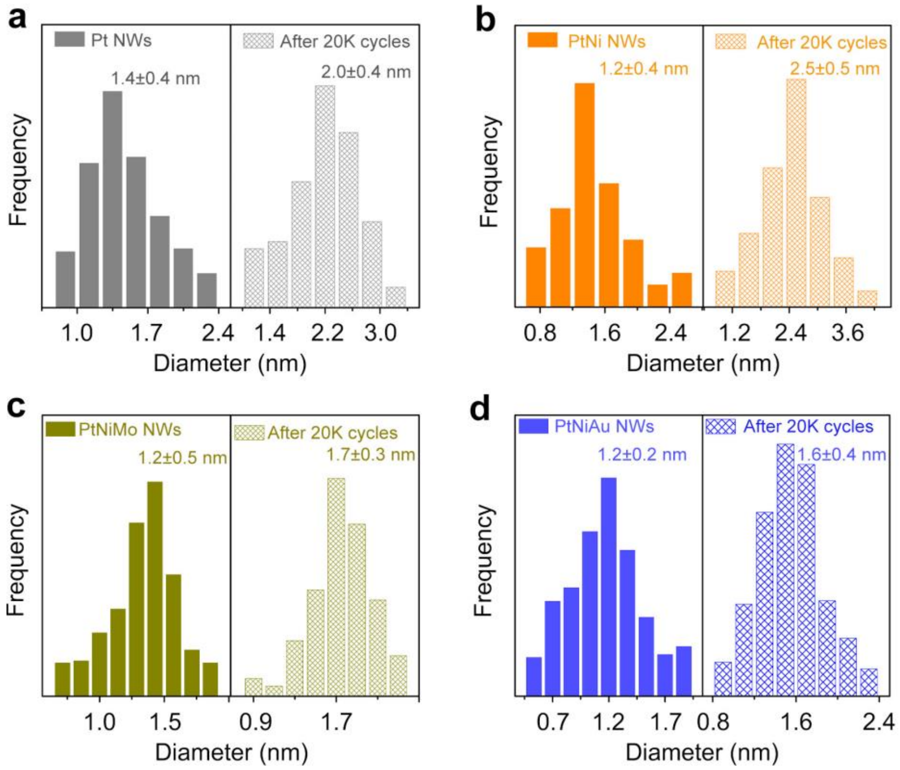

Supplementary Figure 9. The histograms of diameter distributions (a-d) for different catalysts before and after 20K cycles.

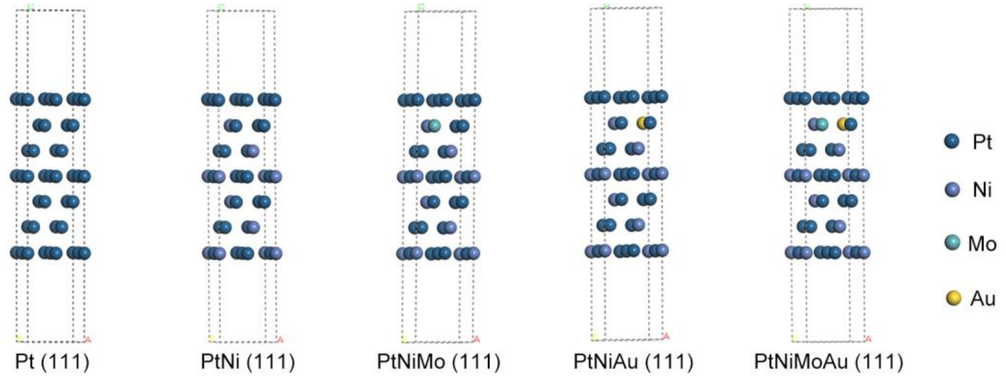

Supplementary Figure 10. The theoretical model structures of PtNi (111), PtNiMo (111), PtNiAu (111) and PtNiMoAu (111) slabs.

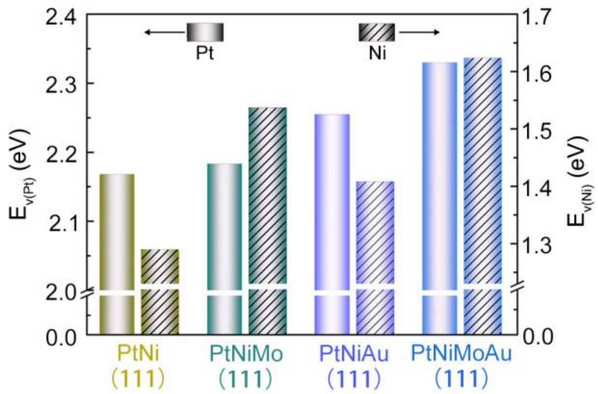

Supplementary Figure 11. Pt and Ni vacancy formation energies ($E_{V(Pt/Ni)}$) for different slabs.

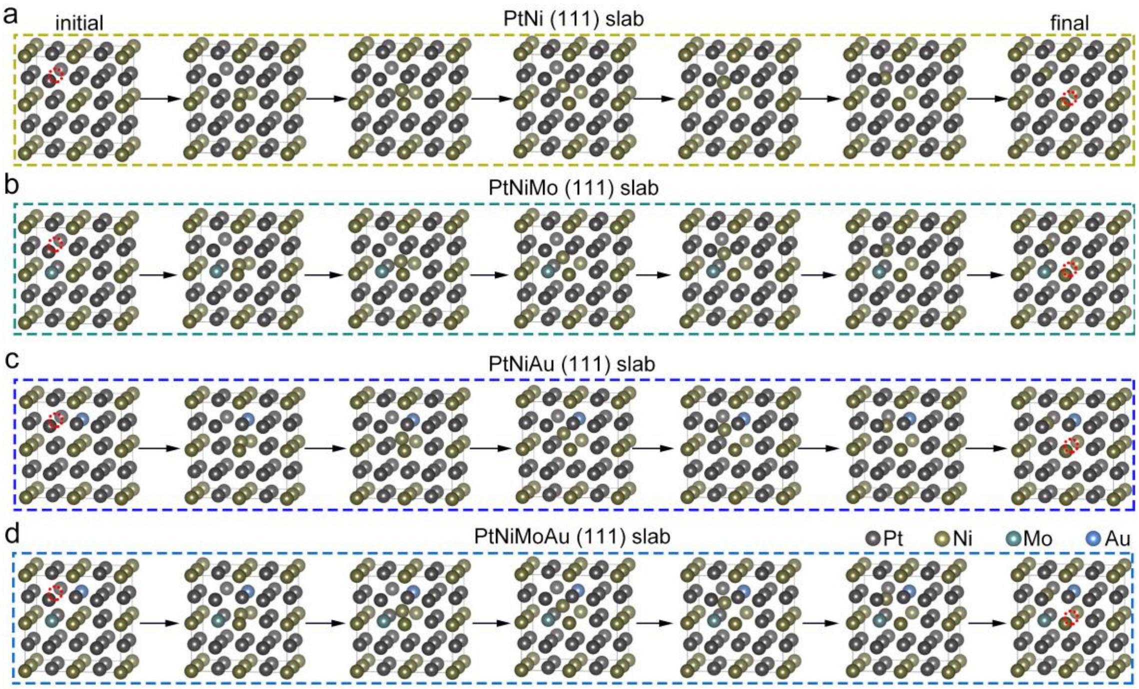

Supplementary Figure 12. Proposed Ni diffusion pathway on a) PtNi (111), b) PtNiMo (111), c) PtNiAu (111) and d) PtNiMoAu (111) slabs.

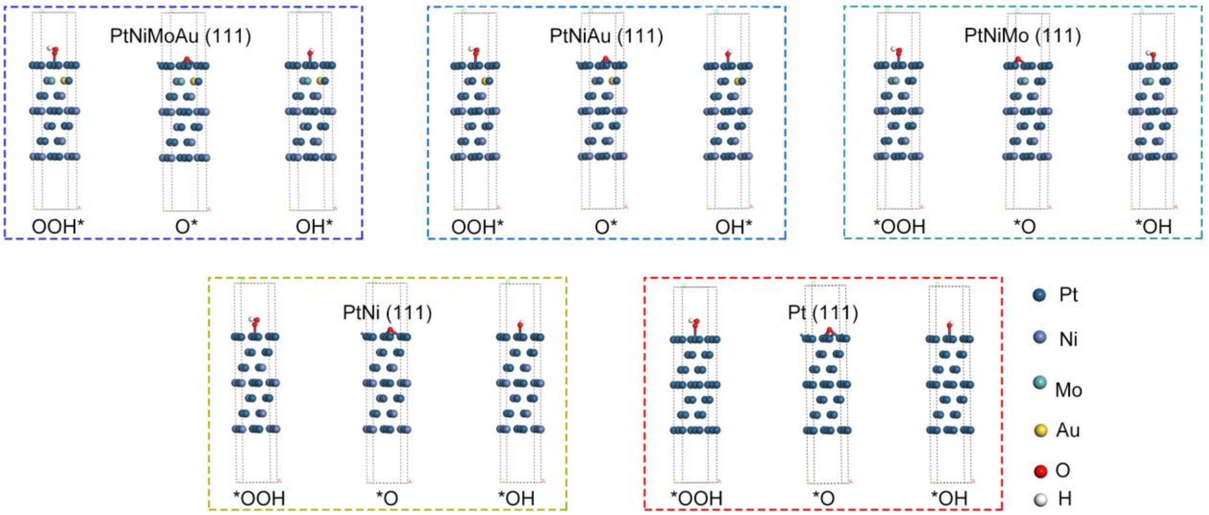

Supplementary Figure 13. The adsorbed intermediate configurations for ORR on PtNiMoAu (111), PtNiAu (111), PtNiMo (111), PtNi (111), and Pt (111) slabs.

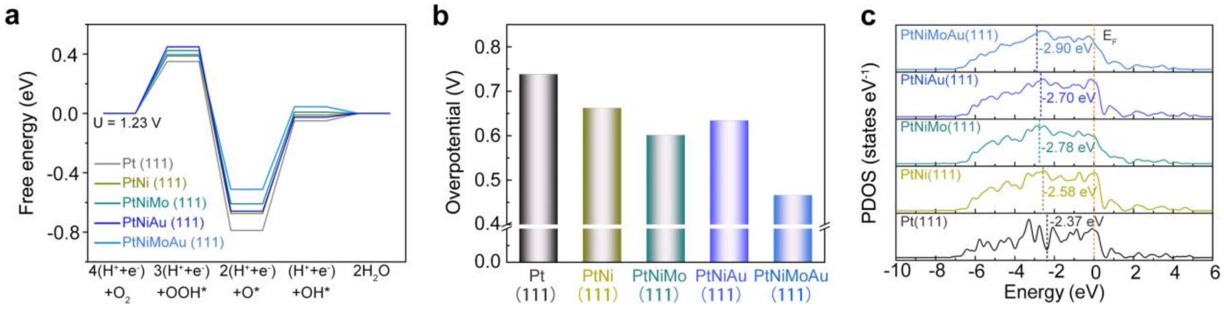

Supplementary Figure 14. a) The calculated free energy diagrams at the equilibrium potential (U=1.23 V). b) The calculated overpotentials for ORR on Pt (111), PtNi (111), PtNiMo (111), PtNiAu (111), and PtNiMoAu (111) slabs. c) The plots of projected *d*-density of states (PDOS) for surface Pt atoms on Pt (111), PtNi (111), PtNiMo (111), PtNiAu (111), and PtNiMoAu (111) slabs.

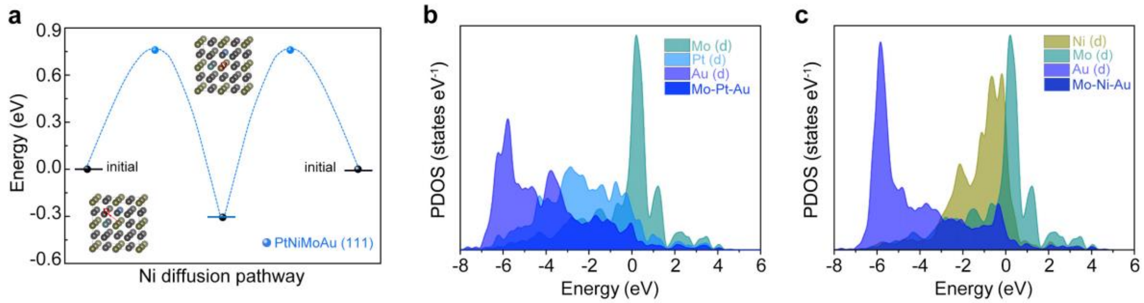

Supplementary Figure 15. a) Ni diffusion pathway energy diagram for PtNiMoAu (111) slab. b) and c) The PDOSs. Site-dependent PDOSs of Pt-5d, Ni-3d, Mo-3d and Au-3d on PtNiMoAu (111) slabs.

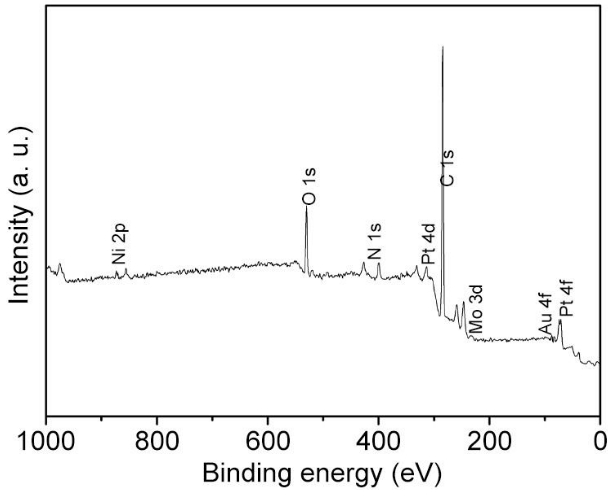

Supplementary Figure 16. The XPS survey spectrum of the PtNiMoAu NWs.

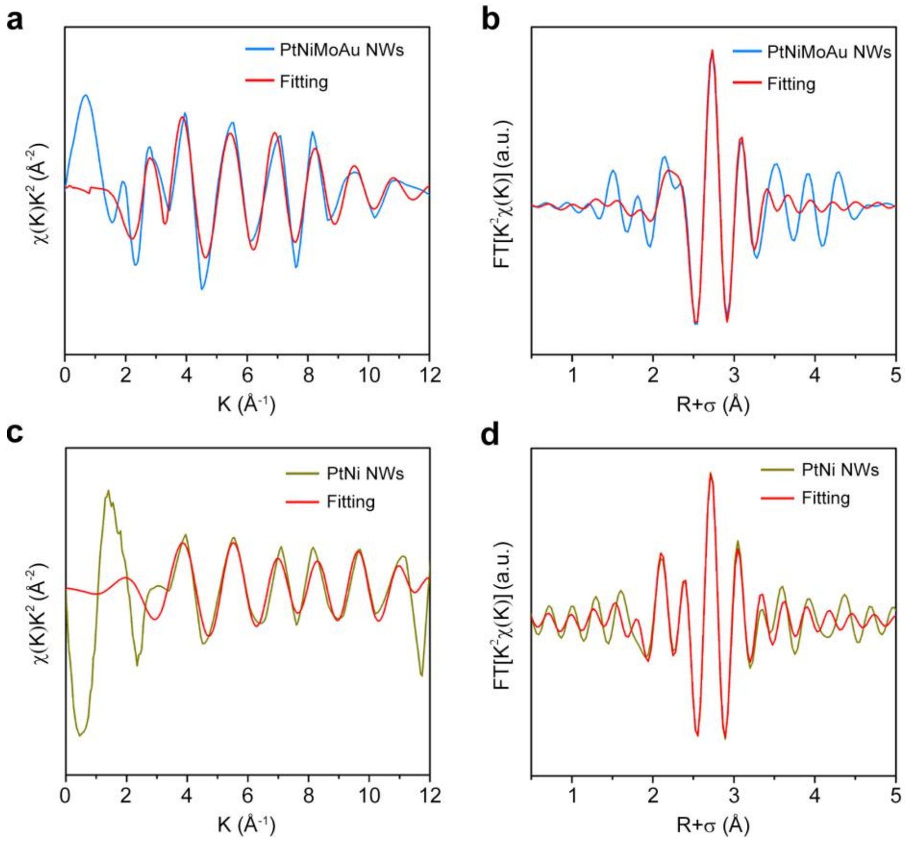

Supplementary Figure 17. EXAFS analysis of a) PtNiMoAu NWs and c) PtNi NWs in K space. First-shell EXAFS fitting b) PtNiMoAu NWs and d) PtNi NWs in R space for spectral data. $FT[k^2\chi(k)]$, Fourier-transformed k^2-weighted EXAFS. The fitting results are shown with red lines.

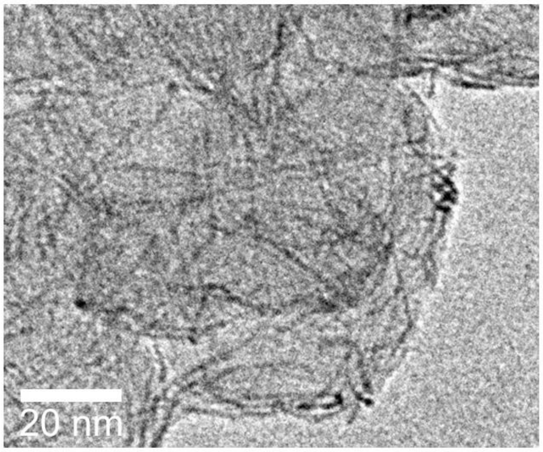

Supplementary Figure 18. TEM images of PtNiMoAu NWs/C catalyst.

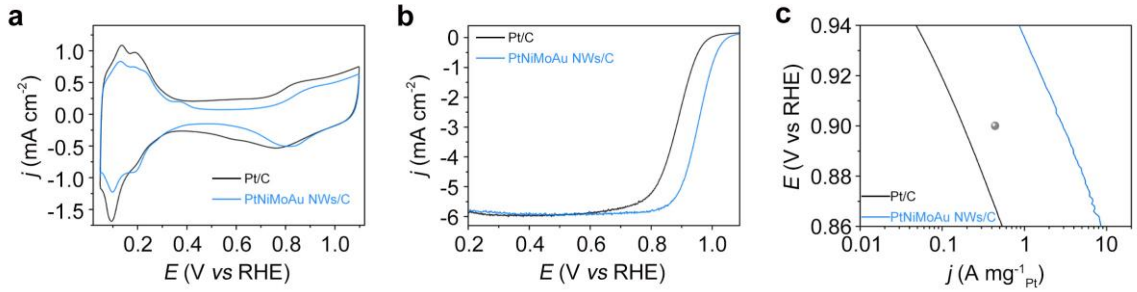

Supplementary Figure 19. a) CVs of PtNiMoAu NWs/C and Pt/C catalysts recorded in N_2-saturated 0.1 M $HClO_4$ solutions at a sweep rate of 50 mV s^{-1}. b) ORR polarization curves of PtNiMoAu NWs/C and Pt/C catalysts recorded in O_2-saturated 0.1 M $HClO_4$ solutions at a sweep rate of 10 mV s^{-1} and a rotation rate of 1600 rpm. c) Tafel plots of mass activity for PtNiMoAu NWs/C and Pt/C catalysts. The Gray ball denotes the mass activity for DOE target (0.44 A mg^{-1}_{Pt}).

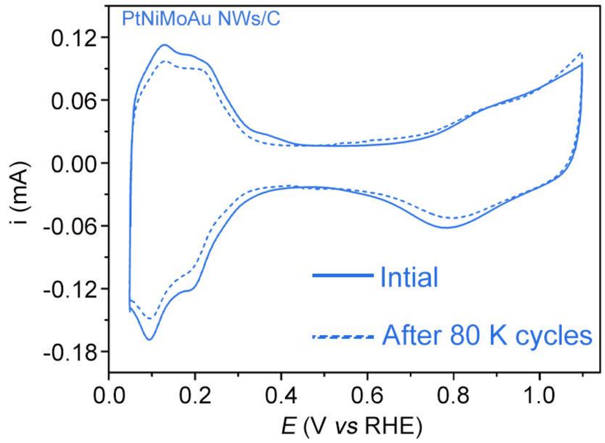

Supplementary Figure 20. Long-term durability test of the commercial PtNiMoAu/C catalyst. CV curves of PtNiMoAu/C before and after 80K cycles of ADT.

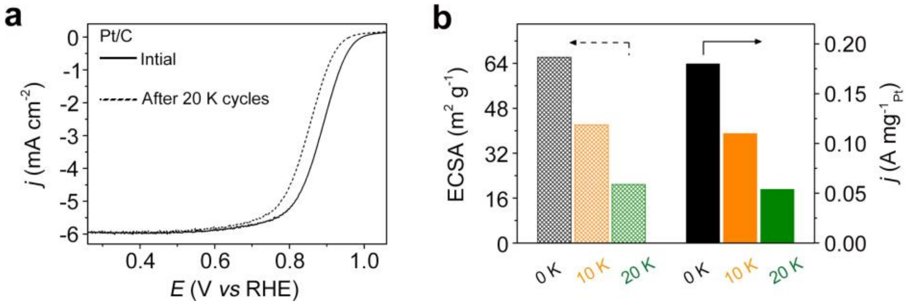

Supplementary Figure 21. Long-term durability test of the commercial Pt/C catalyst. (a) ORR polarization curves of commercial Pt/C before and after 20K cycles of ADT. (b) The changes in ECSA and mass activities of commercial Pt/C before and after different cycles of ADT.

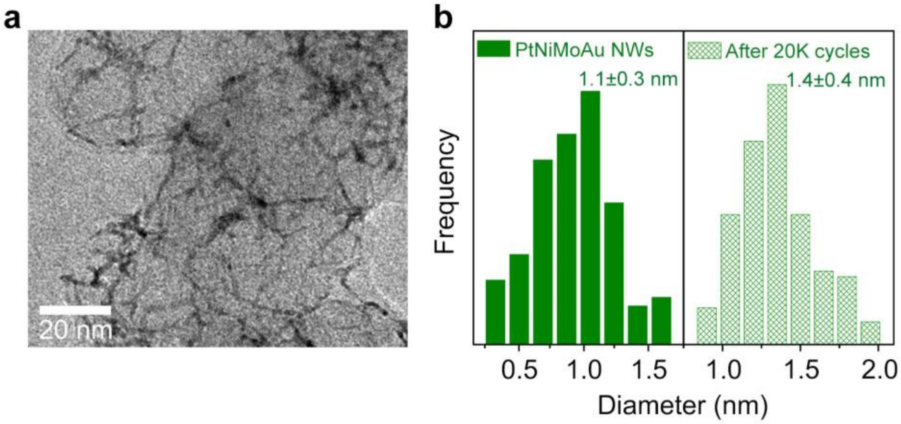

Supplementary Figure 22. a) TEM images of PtNiMoAu NWs/C after 80K cycles. b) The histograms of diameter distributions for PtNiMoAu NWs/C before and after 20K cycles.

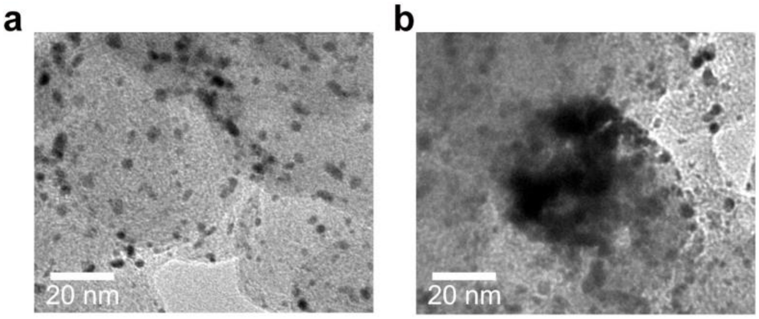

Supplementary Figure 23. TEM images of Pt /C a) before and b) after 20K cycles.

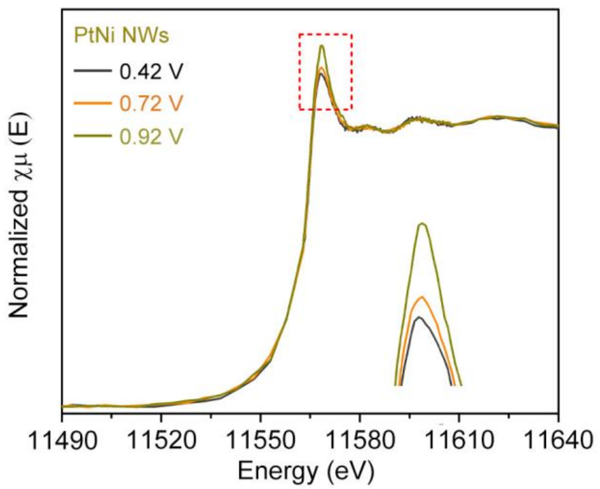

Supplementary Figure 24. In situ Pt L_3-edge XANES spectra of PtNi NWs at different potentials. The inset showed an enlarged view of the area marked by the red rectangle.

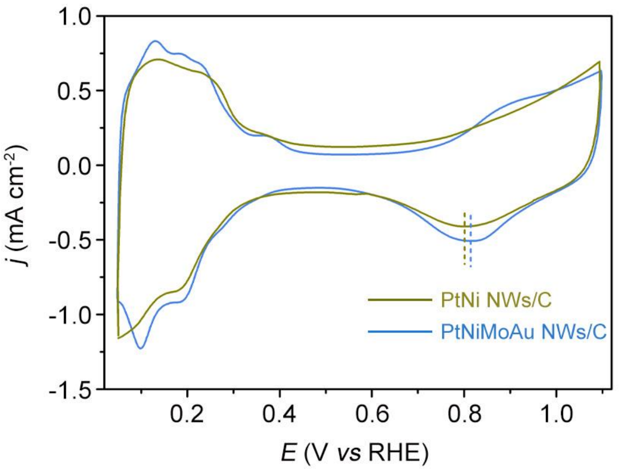

Supplementary Figure 25. CV curves of PtNi NWs/C and PtNiMoAu NWs/C catalysts.

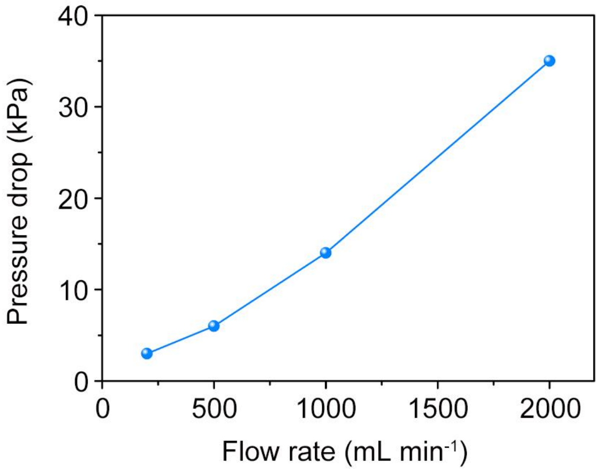

Supplementary Figure 26. The pressure drops between the inlet and outlet at different flow rate.

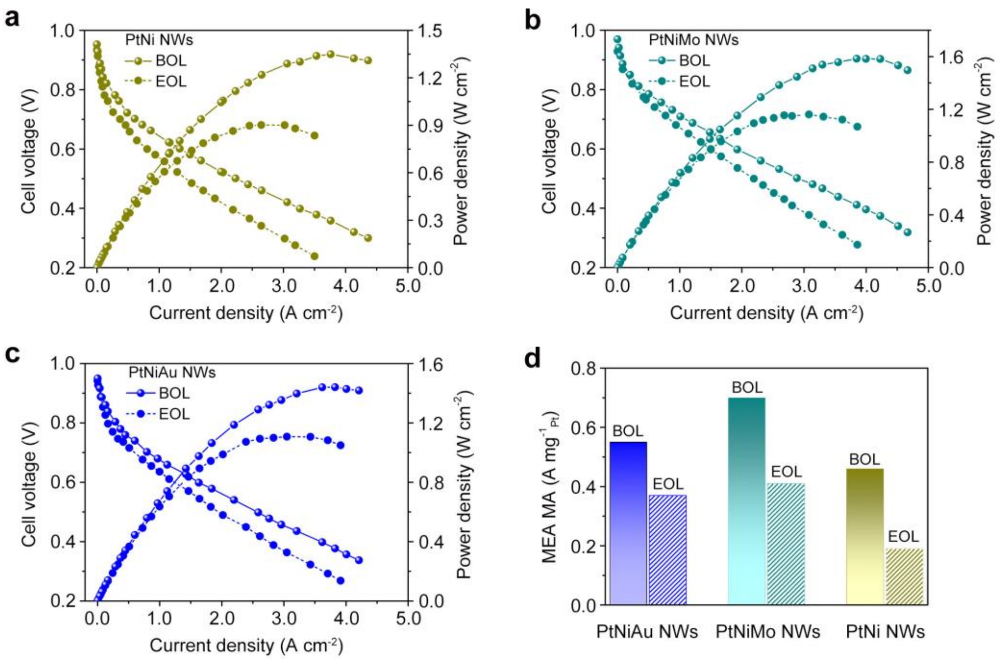

Supplementary Figure 27. H_2-O_2 fuel cell polarization curves and power density curve s of PtNi NWs/C (a), PtNiMo NWs/C (b), and PtNAu NWs/C (c) before and after 30K cycles of ADT. Anode: H_2 flow rate = 200 mL min^{-1}, 0.05 mg$_{Pt}$ cm^{-2} for Pt/C; Cathode: O_2 flow rate = 500 mL min^{-1}, 0.10 mg$_{Pt}$ cm^{-2} for PtNi-based NWs/C. (d) Changes of MA for PtNi NWs/C, PtNiMo NWs/, and PtNAu NWs/C before and after 30K cycles of ADT.

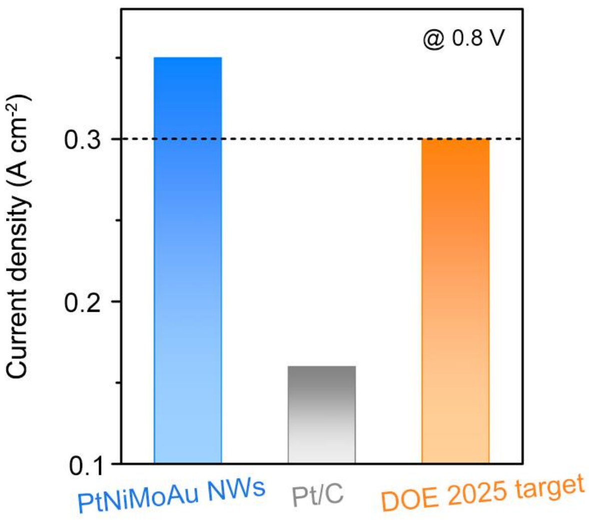

Supplementary Figure 28. Comparisons in current density at 0.8 $V_{iR\text{-}free}$ for PtNiMoAu NWs, Pt/C and DOE 2025 target (H_2-Air fuel cell).

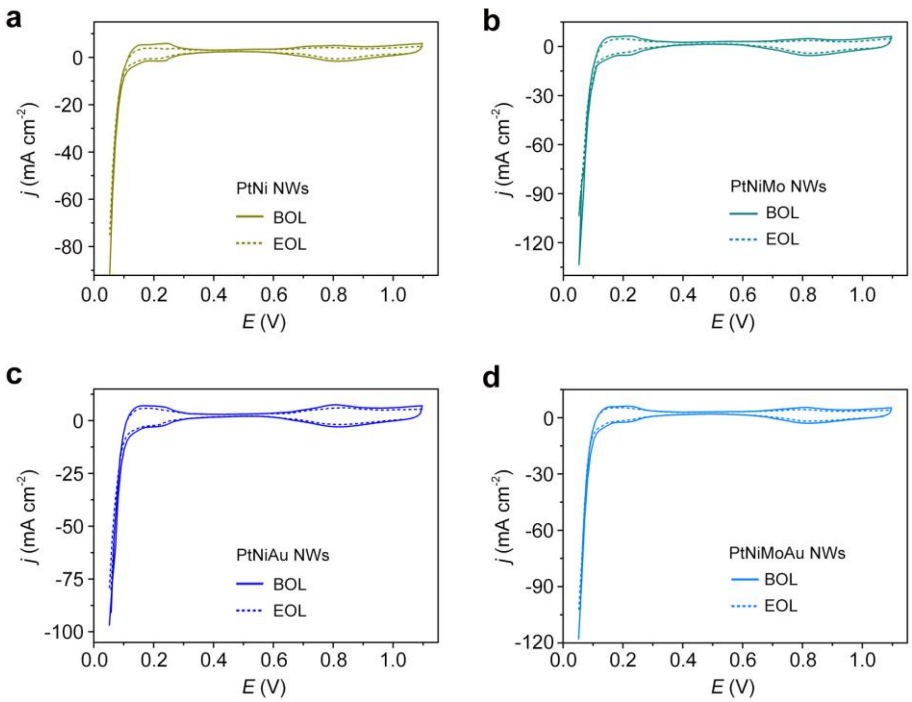

Supplementary Figure 29. CV curves of PtNi NWs/C (a), PtNiMo NWs/C (b), PtNAu NWs/C (c), and PtNMoAu NWs/C (d) before and after 30K cycles of ADT in PEMFCs.

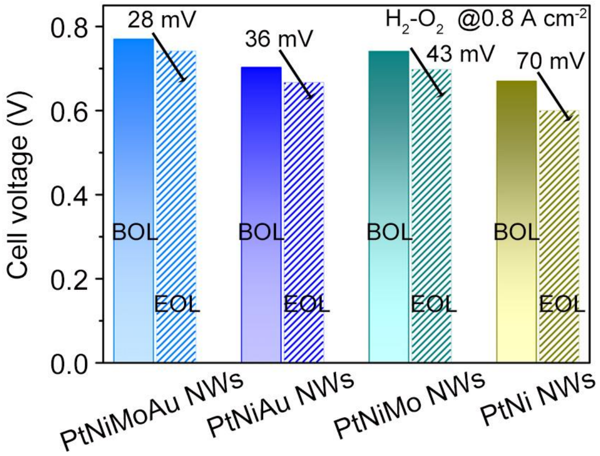

Supplementary Figure 30. The changes of cell voltage (H_2-O_2) at 0.8 A cm^{-2} of PtNiMoAu NWs/C, PtNiAu NWs/C, PtNiMo NWs/C, and PtNi NWs/C before and after 30K cycles of ADT.

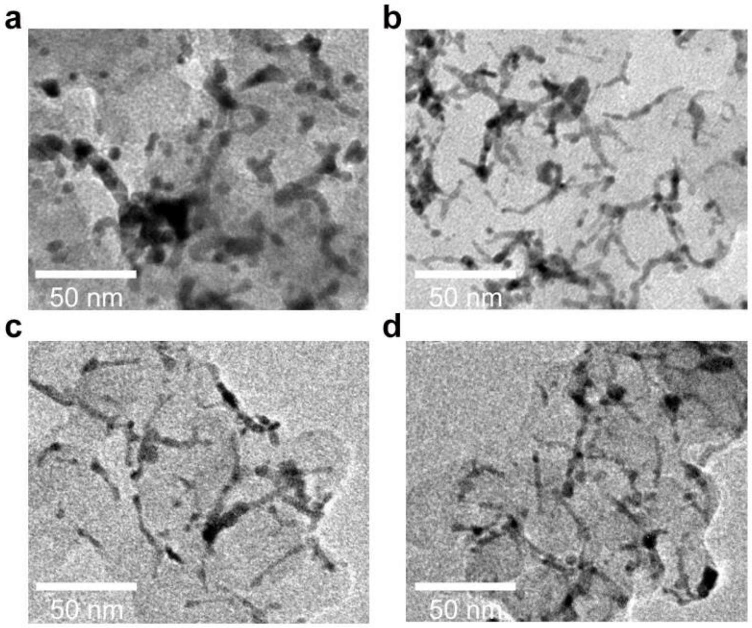

Supplementary Figure 31. TEM images of a) PtNi NWs/C, b) PtNiMo NWs/C, c) PtNiAu NWs/C, and d) PtNiMoAu NWs/C after MEA durability test.

Supplementary Table 1 Synthetic parameters and structural analysis of all ultrathin Pt-based NWs. The other synthesis parameters such as $Pt(acac)_2$ (20.0 mg), CTAB (75 mg), $W(CO)_6$ (20.0 mg), OAm (4 mL) and reaction time (2 h) were kept the same for all ultrathin Pt-based NWs.

	Sample	Synthetic parameters		Structural analysis		
		**Other precursors /mg**	**T/ºC**	**Atomic ratio based on XPS**	**Length /nm**	**Dimeter /nm**
**1**	**Pt NWs**		165		60 ± 22	1.4 ± 0.4
**2**	**PtNi NWs**	$Ni(acac)_2$/8.0	165	Pt/Ni=3.03:1.00	65 ± 22	1.2 ± 0.4
**3**	**PtNiMo NWs**	$Ni(acac)_2$/10.0<br>$Mo(acac)_3$/7.0	170	Pt/Ni/Mo<br>=2.99:1.00:0.10	60 ± 26	1.2 ± 0.5
**4**	**PtNiAu NWs**	$Ni(acac)_2$/10.0<br>$HAuCl_4\cdot 3H_2O$/2.5	170	Pt/Ni/Au<br>=3.01:1.00:0.09	62 ± 25	1.2 ± 0.2
**5**	**PtNiMoAu NWs**	$Ni(acac)_2$/10.0<br>$Mo(acac)_3$/8<br>$HAuCl_4\cdot 3H_2O$/2.7	175	Pt/Ni/Mo/Au=<br>3.02:1.00:0.11:0.13	62 ± 21	1.1 ± 0.3

Supplementary Table 2. RDE performance comparisons for PtNiAu NWs/C, PtNiMo NWs/C, PtNi NWs/C, and Pt NWs/C catalysts in this work.

	**Catalysts**	**Mass activity /A mg_{Pt}^{-1}**	**ECSA /m^2 g^{-1}**	**Specific activity /mA cm^{-2}**
**1**	**Pt NWs**	0.84	75.5	1.11
**2**	**PtNi NWs**	1.96	83.8	2.33
**3**	**PtNiMo NWs**	2.54	86.7	2.93
**4**	**PtNiAu NWs**	2.24	85.7	2.61

Supplementary Table 3. Bader analysis for PtNi (111) and PtNiMoAu (111) slabs.

**Slabs**	**PtNi (111)**	**PtNiMoAu (111)**
Average charge of Pt (e)	0.094	0.197

Supplementary Table 4. Structural parameters extracted from the quantitative EXAFS curve fitting.

Catalyst	Path	*R* (Å)	*N*	ΔE_0 (eV)	σ^2 (Å^2)	*R*-factor
**Pt foil**	Pt-Pt	2.76±0.01	12	7.9	0.003±0.001	0.004
**PtNi NWs**	Pt-Pt/Ni	2.72±0.03	6.3±0.4	3.9	0.004±0.001	0.007
**PtNiMoAu NWs**	Pt-Pt/Ni/Mo/Au	2.70±0.02	5.5±0.4	2.2	0.006±0.002	0,009

R, bond length; *N*, coordination number; ΔE_0, inner potential shift; σ^2, Deby-Waller factor.

Supplementary Table 5. RDE performance comparisons for PtNiMoAu NWs/C catalyst in this work with the representative catalysts from recent works. NA, not available.

Catalysts	Degradation of MA after ADT		Test conditions (temperature, potential range, sweep rate)	Ref.
	Loss (%)	Cycles		
**PtNiMoAu NWs/C**	1.7	10K	room temperature, 0.6~1.0 V_{RHE}, 100 mV s^{-1}	This work
**PtNiMoAu NWs/C**	7.6	40K	room temperature, 0.6~1.0 V_{RHE}, 100 mV s^{-1}	This work
**PtNiMoAu NWs/C**	16.2	80K	room temperature, 0.6~1.0 V_{RHE}, 100 mV s^{-1}	This work
**Pt_3Ni-NCs**	5.1	10K	room temperature, 0.6~1.1 V_{RHE}, 100 mV s^{-1}	1
**$L1_0$-W-PtCo**	7.7	10K	room temperature, NA, 100 mV s^{-1}	2
**Fct-PtFeIr**	9.8	10K	room temperature, NA, 100 mV s^{-1}	3
**PtNiRh NWs/C**	12.8	10K	room temperature, 0.6~1.1 V_{RHE}, 100 mV s^{-1}	4
**sd-$Pt_{84}Fe_{12}Co_4$**	12.0	20K	room temperature, 0.6~1.1 V_{RHE}, 100 mV s^{-1}	5
**Re-PtNiGa NWs**	10.8	20K	room temperature, 0.6~1.1 V_{RHE}, 100 mV s^{-1}	6
**$Pt_{4.31}Ga$ NWs/C**	15.8	30K	room temperature, 0.6~1.1 V_{RHE}, 100 mV s^{-1}	7
**$L1_0$-PtCo/Pt**	18.0	30K	room temperature, 0.6~1.1 V_{RHE}, 100 mV s^{-1}	8
**$Pt_{24}Fe_{76}$ NWs/C**	17.1	40K	room temperature, 0.6~1.0 V_{RHE}, 100 mV s^{-1}	9
**Pt_3Fe z-NWs/C**	24.6	50K	room temperature, 0.6~1.1 V_{RHE}, NA	10

Supplementary Table 6. The changes of atomic ratio for PtNiMoAu NWs/C, PtNiAu NWs/C, PtNiMo NWs/C, and PtNi NWs/C before and after 30K cycles of MEA.

Catalysts			Pt	Ni	Mo	Au
**1**	**PtNi NWs**	BOL	3.03	1.00	-	-
		EOL	14.78	1.00	-	-
**2**	**PtNiMo NWs**	BOL	2.99	1.00	0.10	-
		EOL	6.98	1.00	0.21	-
**3**	**PtNiAu NWs**	BOL	3.01	1.00	-	0.11
		EOL	8.22	1.00	-	0.19
**4**	**PtNiMoAu NWs**	BOL	3.00	1.00	0.11	0.13
		EOL	5.09	1.00	0.16	0.15

Supplementary Table 7. Fuel-cell performance for PtNiMoAu NWs/C catalyst and the representative results from recent works.

**Catalysts**	**BOL MA (A mg_{Pt}^{-1}, 0.9 V)**	**MA retention (%)**	**Ref.**
**PtNiMoAu NWs/C**	**0.93**	**77.4**	**This work**
**$L1_0$-Pt_2CuGa/C**	0.57	64.9	11
**Pt_3FeCo NSs/C**	0.79	67.0	12
**i-CoPt@Pt/KB**	0.53	64.5	13
**$Pt_{1.5}Ni_{1-x}$/Ni–N–C**	0.70	73.7	14
**Pt_3Co/FeN_4-C**	0.72	62.0	15
**Pt-Co UHT**	0.46	83.0	16
**$PtNi/C_3$**	0.66	65.0	17
**PtCo@Gnp**	1.21	73.0	18
**PtCo i-NP**	1.27	79.0	19
**LP@PF-1**	1.08	64.0	20

Supplementary References

(1) H. Ding, P. Wang, C. Su, H. Liu, X. Tai, N. Zhang, H. Lv, Y. Lin, W. Chu, X. Wu, C. Wu, Y. Xie, *Adv. Mater.* **2022**, *34*, 2109188.

(2) J. Liang, N. Li, Z. Zhao, L. Ma, X. Wang, S. Li, X. Liu, T. Wang, Y. Du, G. Lu, J. Han, Y. Huang, D. Su, Q. Li, *Angew. Chem., Int. Ed.*, **2019**, *131*, 15617.

(3) Z. Yang, H. Yang, L. Shang, T. Zhang, *Angew. Chem., Int. Ed.*, **2022**, *61*, e202113278.

(4) K. Li, X. Li, H. Huang, L. Luo, X. Li, X. Yan, C. Ma, R. Si, J. Yang, J. Zeng, *J. Am. Chem. Soc.*, **2018**, *140*, 16159.

(5) J. Huang, L. Sementa, Z. Liu, G. Barcaro, M. Feng, E. Liu, L. Jiao, M. Xu, D. Leshchev, S.-J. Lee, M. Li, C. Wan, E Zhu, Y. Liu, B. Peng, X. Duan, W. A. Goddard III, A. Fortunelli, Q. Jia, Y. Huang, *Nat. Catal*. **2022**, *5*, 513.

(6) L. Gao, T. Su, X. Tan, M. Liu, F. Xue, B. Wang, J. Zhang, Y.-F. Lu, C. Ma, H. Tian, S. Yang, S. C. Smith, H. Huang, *Appl. Catal. B: Environ*. **2022**, *303*, 120918.

(7) L. Gao, X. Li, Z. Yao, H. Bai, Y. Lu, C. Ma, S. Lu, Z. Peng, J. Yang, A. Pan and H. Huang, *J. Am. Chem. Soc.*, **2019**, *141*, 18083.

(8) J. Li, S. Sharma, X. Liu, Y.-T. Pan, J. S. Spendelow, M. Chi, Y. Jia, P. Zhang, D. A. Cullen, Z. Xi, H. Lin, Z. Yin, B. Shen, M. Muzzio, C. Yu, Y. S. Kim, A. A. Peterson, K. L. More, H. Zhu, S. Sun, *Joule* **2019**, *3*, 124.

(9) Z. Kong, Y. Maswadeh, J. A. Vargas, S. Shan, Z.-P. Wu, H. Kareem, A. C. Leff, D. T. Tran, F. Chang, S. Yan, S. Nam, X. Zhao, J. M. Lee, J. Luo, S. Shastri, G. Yu, V. Petkov and C.-J. Zhong. *J. Am. Chem. Soc*., **2020**, *142*, 1287−1299.

(10) M. Luo, Y. Sun, X. Zhang, Y. Qin, M. Li, Y. Li, C. Li, Y. Yang, L. Wang, P. Gao, G. Lu and S. Guo, *Adv. Mater.*, **2018**, *30*, 1705515.

(11) X. Liu, Z. Zhao, J. Liang, S. Li, G. Lu, C. Priest, T. Wang, J. Han, G. Wu, X. Wang, Y. Huang, Q. Li, *Angew. Chem., Int. Ed.*, **2023**, 10.1002/anie.202302134.

(12) L. Bu, J. Liang, F. Ning, J. Huang, B. Huang, M. Sun, C. Zhan, Y. Ma, X. Zhou, Q. Li, X. Huang, *Adv. Mater.* **2022**, *35*, 2208672.

(13) T. Y. Yoo, J. Lee, S. Kim, M. Her, S.-Y. Kim, Y.-H. Lee, H. Shin, H. Jeong, A. K. Sinha, S.-P. Cho, Y.-E. Sung, T. Hyeon, *Energy Environ. Sci.* **2023**, *16*, 1146.

(14) W. Guo, X. Gao, M. Zhu, C. Xu, X. Zhu, X. Zhao, R. Sun, Z. Xue, J. Song, L. Tian, J. Xu, W. Chen, Y. Lin, Y. Li, H. Zhou, Y. Wu, *Energy Environ. Sci.* **2023**, *16*, 148.

(15) Z.Qiao, C. Wang, C. Li, Y. Zeng, S. Hwang, B. Li, S. Karakalos, J. Park, A. J. Kropf, E. C. Wegener, Q. Gong, H. Xu, G. Wang, D. J. Myers, J. Xie, J. S. Spendelow, G. Wu, *Energy Environ. Sci.* **2021**, *14*, 4948.

(16) J. Liu, S. Liu, F. Yan, Z. Wen, W. Chen, X. Liu, Q. Liu, J. Shang, R. Yu, D. Su, J. Shui, *J. Am. Chem. Soc*. **2022**, *144*, 19106.

(17) Z. Zhao, M. D. Hossain, C. Xu, Z. Lu, Y.-S. Liu, S.-H. Hsieh, I. Lee, W. Gao, J. Yang, B. V. Merinov, W. Xue, Z. Liu, J. Zhou, Z. Luo, X. Pan, F. Zaera, J. Guo, X. Duan, W. A. Goddard III, Y. Huang, *Matter* **2020**, *3*, 1774.

(18) Z. Zhao, Z. Liu, A. Zhang, X. Yan, W. Xue, B. Peng, H. L. Xin, X. Pan, X. Duan, Y. Huang, *Nat. Nanotech*. **2022**, *17*, 968.

(19) C.-L. Yang, L.-N. Wang, P. Yin, J. Liu, M.-X Chen, Q.-Q. Yan, Z.-S. Wang, S.-L. Xu, S.-Q. Chu, C. Cui, H. Ju, J. Zhu, Y. Lin1, J. Shui, H.-W. Liang, *Science* **2021**, *374*, 459.

(20) L. Chong, H. Barkholtz, W. Ding, J. Wen, J. Kubal, F. G. Sen, J. Zou, D.-J. Liu, *Science* **2018**, *362*, 1276.
